# Examining outcomes for service users accessing the Breaking Free Online computer-assisted therapy program for substance use disorders via a ‘telehealth’ approach: protocol for a two arm, parallel group randomized controlled trial

**DOI:** 10.1186/s13722-023-00391-0

**Published:** 2023-06-02

**Authors:** Sarah Elison-Davies, Lauren Pittard, Tracey Myton, Andrew Jones, Jonathan Ward, Glyn Davies

**Affiliations:** 1 TELUS Health, Williams House, Manchester Science Park, Lloyd Street North, Manchester, M15 6SE UK; 2 Greater Manchester Mental Health NHS Foundation Trust, Achieve Bolton, 69–73 Manchester Road, Bolton, BL2 1ES UK; 3 Faculty of Biology, Medicine and Health, University of Manchester, Oxford Road, Manchester, M15 6JA UK

**Keywords:** Substance use disorders, Computer-assisted therapy, Digital, Cognitive behavioral therapy, Recovery, Telehealth

## Abstract

**Background:**

Breaking Free Online (BFO), a computer-assisted therapy (CAT) program for substance use disorders (SUD), has been available across UK treatment services for the past decade and has demonstrated efficacy. The Covid-19 pandemic has contributed to digital and ‘telehealth’ approaches to healthcare delivery becoming more common and accepted, and has in parallel, increased numbers of referrals to SUD services because of the impact pandemic-related stress has had on substance using habits in the general population. Digital and telehealth approaches, such as BFO, have the potential to support the treatment system to meet this increased demand for SUD services.

**Methods:**

Parallel-group randomized controlled trial of eight-week BFO as an adjunct to standard treatment for SUD, in comparison to standard treatment only, at a National Health Service (NHS) Mental Health Trust in North-West England. Participants will be service users aged 18 years and over with demonstrable SUD for at least 12-months. Interventional and control groups will be compared on multiple measures from baseline to post-treatment assessment at eight-weeks, and then three and six-months follow-up. Primary outcome will be self-reported substance use, with secondary outcomes being standardized assessments of substance dependence, mental health, biopsychosocial functioning and quality of life.

**Discussion:**

This study will examine whether BFO and telehealth support, when delivered as an adjunct to standard SUD interventions, improves outcomes for services users receiving NHS SUD treatment. Findings from the study will be used to inform both developments to the BFO program and guidance around augmenting the delivery of CAT programs via telehealth.

*Trial registration* registered with ISRCTN on 25th May 2021—registration number: 13694016. Protocol version: 3.0 05th April 2022. Trial status: This trial is currently open to recruitment—estimated to be completed in May 2023.

## Introduction

Computer-assisted therapies (CAT) provide access to interventions, such as cognitive-behavioral therapy (CBT), via the internet. CAT can widen access to treatment, be more cost-effective than in-person therapy [1, 2] and optimize treatment fidelity [3]. Meta-analyses demonstrate CAT to be as effective at reducing self-reported levels of anxiety and depression as standard in-person treatments, and superior to waiting list and active control conditions [4–7]—subsequently, CAT is recommended by the UK National Institute for Health and Care Excellence (NICE) [8].

The evidence-base for CAT for substance use disorders (SUD) has been growing for over a decade e.g. [9–11]. Accessing interventions online may overcome specific barriers to accessing SUD services including shame and stigma [12]. Breaking Free Online (BFO) is a tailorable CAT program to support recovery from SUD, which has been delivered via UK community services for the past 12-years, and in UK criminal justice services for the past eight years. And since 2019, BFO has been delivered in both Canadian community and US correctional settings. BFO can be delivered as a ‘self-help’ program or as a structured practitioner-facilitated program and contains 12 core evidence-based ‘behavioral change techniques’ (BCTs) [13], which are informed by CBT [14], relapse prevention [15], mindfulness [16], and motivational enhancement [17]. BFO has been designed to complement and augment standard treatments for SUD such as key-working, counselling and medications, and due to its digital nature, can extend access to evidence-based interventions outside of usual treatment service operating hours, which are usually Monday through Friday, between the hours of 9am and 5pm.

Research around the efficacy of BFO has demonstrated significant reductions in substance use, substance dependence, depression and anxiety, and biopsychosocial impairment, and improvements in quality of life—these studies have been conducted with people using opiates [18], cannabis [19], alcohol [20] and methamphetamine [21]. BFO studies have also been conducted in multiple treatment settings, including eTherapy ‘dual diagnosis’ mental health services [22, 23] and in UK [24, 25] and US [21] prisons. Research has also indicated that users follow tailoring advice provided by the program [26] and has also demonstrated a ‘dose response’ [19, 25, 26]. Baseline clinical characteristics of individuals engaging with the program have been demonstrated to be associated with engagement and outcomes [18, 20, 27].

A significant barrier to accessing in-person SUD services since March 2020 has been the impact of societal lockdowns to curb transmission of ‘severe acute respiratory syndrome coronavirus-2’ (SAR-CoV-2), or ‘COVID-19’. Social isolation throughout the pandemic, a known risk factor for SUD [28], may not have only potentially increased the vulnerability of individuals already living with SUD, but also increased the numbers of people with SUD. Figures published by the charity Alcohol Change UK indicate that COVID-19 has had a detrimental effect on drinking habits in the UK, with one in five individuals reporting increased alcohol consumption after the lockdown measures were introduced—this represents 8.6 million adults [29]. Studies conducted during the pandemic indicate that this increased consumption is associated with greater depression, anxiety and stress [30].

Despite the relative success of the COVID-19 vaccine roll-out in the UK, and the subsequent easing of social distancing restrictions, healthcare services appear to have been transformed for the long-term as a result of the pandemic, with providers continuing to use remote digital and telehealth approaches to deliver their services going forward [31]. This, alongside the increased numbers of people presenting to SUD treatment services because of changing substance use habits during the pandemic, mean research into the ability of digital treatment to effectively meet treatment demand is timely. A systematic review conducted before the COVID-19 pandemic demonstrated that when SUD services are delivered via telehealth approaches, they can provide an effective alternative to in-person services, especially during times when access to services is limited [32]. In addition, a study conducted during the pandemic has suggested that SUD services found telehealth approaches relatively easy to implement when in-person services were restricted, and that these approaches were important in ensuring service users could continue to access services [33]. Additionally, previous research into the efficacy of using a telehealth approach to deliver BFO prior to the pandemic suggested significant improvements in mental health and social functioning [22, 23].

This research highlights the need for substance use interventions that are able to (i) target novel illicit substance use, which may continue to rise as the pandemic runs its course, (ii) be delivered in ways that do not rely on service users accessing them via in-person appointments, which may be particularly important given the increased demand for services due to the impact of the pandemic, (iii) be rapidly upscaled in order to meet the increasing numbers of individuals seeking help for their substance use, and (iv) address the concurrent mental health difficulties that often occur alongside substance use. BFO has therefore been identified as a potential adjunct to standard treatment that may satisfy these unmet needs.

### Objectives

This study will examine the efficacy of BFO when delivered as an adjunct to standard treatment—BFO will be delivered via a telehealth model in which participants accessing the program will receive fortnightly ‘recovery check-in’ phone calls from their SUD treatment service. Outcomes for this interventional group will be compared to those of a control group receiving standard treatment only.

## Methods

### Trial design

Randomized, open-label, parallel-group, longitudinal randomized controlled superiority trial of (i) BFO delivered via a telehealth model plus standard treatment (intervention group), versus (ii) standard treatment only (active control group). This will be a comparison study of eight-week treatment periods in both the interventional and control groups.

### Study setting

This study will be conducted at a single site within an NHS Mental Health Trust in North-West England. This Trust serves approximately 53,000 service users a year, and the clinical site where the study will be conducted usually receives approximately 2400 referrals of service users with SUD annually, with approximately 1500 of these referrals receiving structured treatment. The Trust provides a range of services for people with SUD including six rehabilitation units, community outreach and young person teams, and a citywide Recovery Pathways service.

### Participants

Participants will be service users currently in treatment for SUD who meet the following inclusion criteria: (i) aged 18 years or above, (ii) experiencing problem alcohol and/or drug use for ≥ 12 months requiring treatment, as determined by clinical personnel at the service, (iii) willing to comply with an eight-week treatment program for problem alcohol and/or drug use, (iv) willing to provide outcome measures post-treatment, and at three and six-months follow-up, (v) able to read, write and communicate in the English language, (vi) willing and able to access an internet enabled device for the eight-week treatment period, (vii) willing and able to give informed consent for participation in the study, and capable of understanding and complying with protocol requirements.

Exclusion criteria are as follows: (i) under 18 years of age, (ii) participation in any other alcohol and/or drug related clinical studies within 12 months prior to date of consent, (iii) detention under the Mental Health Act, (iv) clinically significant intellectual or developmental disability which may impair ability to engage with BFO and/or complete the necessary assessment measures included in the methodology, (v) pregnancy (as self-reported), (vi) previous use of BFO to address drug or alcohol use within the past 12-months.

The projected sample size will require 61 evaluable participants in each of the two treatment groups (interventional and control) to achieve enough observed power (assuming an observed power of 0.80 with α = 0.05) with an allowance of 50% attrition at three and six-months follow-up, in order to detect a medium effect size (d ≤ 0.50). Therefore, to obtain a total of 122 evaluable participants, it is estimated that a total of 183 participants will need to be recruited and screened. These estimations have been based on previous alcohol and drug studies size samples [8], some of which have used longitudinal statistical analyses.

### Recruitment

Participants will be recruited from Greater Manchester Mental Health NHS Foundation Trust’s (GMMH’s) patient population. GMMH practitioners and clinicians will identify potential participants who may meet trial inclusion criteria, and potential participants will be provided with an approved Patient Information Sheet. If the individual wishes to enroll in the trial, a member of the research team will obtain consent from the individual, at which point eligibility will be determined prior to randomization. A member of the research team within the participating organization who is qualified by education, training or experience, will conduct informed consent procedures.

### Interventions

Participants in the intervention group will be encouraged to spend one-hour per week working on BFO for the eight-week treatment period. Online access to BFO is granted via the activation of an online account using a ‘service code’ provided by the study team. Participants must agree to a Terms and Conditions of Use when they activate their account, which is in accordance with the Participant Information Sheet and Informed Consent Form and conforms to the United Kingdom General Data Protection Regulation (GDPR).

**Fig. 1:**
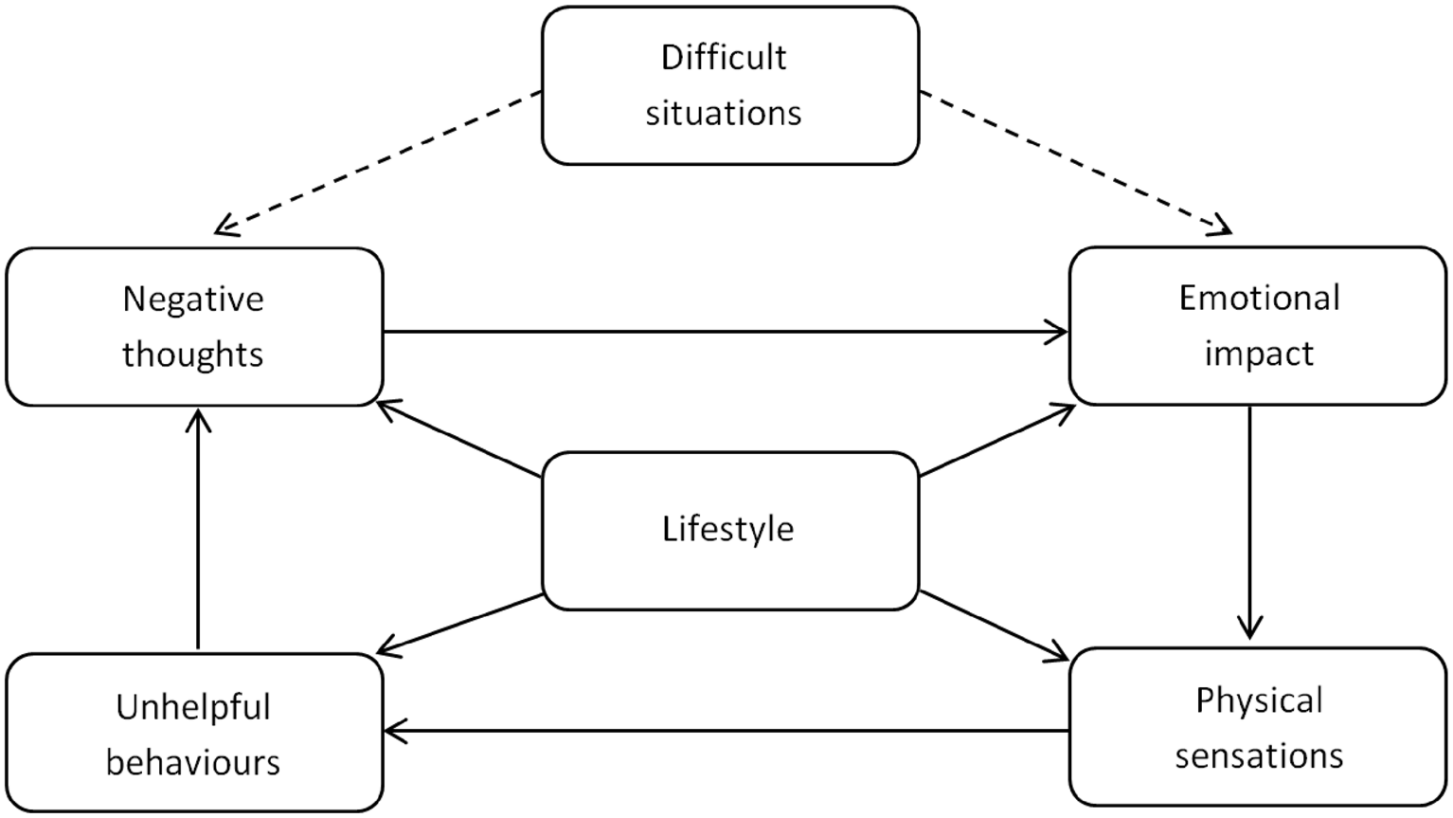
The Lifestyle Balance Model

Upon account activation, each participant will be required to complete a baseline assessment of their substance use and dependence, and their wider biopsychosocial functioning. Included in this assessment is the ‘Recovery Progression Measure’ RPM: [34, 35], which measures levels of functioning across six biopsychosocial domains. BFO then uses these data to populate a six-domain model (see Fig. 1), the ‘Lifestyle Balance Model’ LBM: [36]. The LBM acts as a clinical formulation to help the user understand the specific issues and domains of functioning that may be implicated in their substance use and provides access to the clinical content of the program (See Table 1).

Based on RPM scores, each of the domains of the LBM are colored either green, amber, or red, indicating respectively, ‘little’, ‘moderate’ or ‘significant’ impairment. Tailoring advice then guides the user to complete clinical content of the program that is able to address the domains of their functioning in the LBM where they may be experiencing the greatest levels of impairment (amber and red domains of the LBM). Individuals are able to address these domains of functioning by completing the 12 main BCTs included in the program (see Table 1). Every two-weeks BFO prompts each user to complete a mandatory ‘Progress Check’ assessment, which is comprised of the same items as the baseline assessment. Alongside providing access to the BFO program, participants will receive fortnightly 15–20 min recovery check-in telephone calls from the SUD service—a total of four telephone calls during the treatment period. Phone calls will include questions relating to the participant’s understanding and perception of the content of BFO, their ability to practice and rehearse the skills they are learning from the program, and support needs.

**Table 1:** The 'behavioral change techniques' (BCTs) contained within Breaking Free Online

Content in Breaking Free Online	Description of strategy	Therapeutic approaches underpinning strategies	BCT taxonomy (V1) techniques (number in taxonomy)
Baseline and progress check assessments	Monitor behavior to provide feedback about progress towards goals; Encourage new behaviors via positive feedback	Goal setting; self-monitoring	Self-monitoring of behavior (2.3); Feedback on outcome(s) of behavior (2.7)
Lifestyle Balance Model	Generic formulation; Idiosyncratic formulation; Personalized feedback; Case formulation—understand the links between situations, thoughts, emotions, behaviors, physical sensations, and lifestyle	Node-link mapping (International Treatment Effectiveness Project (ITEP); Cognitive-behavioral therapy (CBT)	Information about antecedents (4.2); Information about health consequences (5.1); Salience of consequences (5.2); Information about social and environmental consequences (5.3); Information about emotional consequences (5.6)
Difficult situations domain of LBM	Assessment; Self-monitoring; Standardized measures; Psycho-education on impact of problematic situations; Intervention to help people in distress access support; Recognize–avoid–cope; Relapse prevention for coping with environmental/situational/emotional triggers; Creating action plans on how to avoid or cope in high risk situations	All structured therapeutic approaches; Psychoeducation; Guided self-help; Relapse prevention; Refusal skills	Social support (unspecified) (3.1); Reduce negative emotions (11.2); Problem solving (1.2); Action planning (1.4); Instruction on how to perform the behavior (4.1); behavioral practice/rehearsal (8.1); behavior substitution (8.2); Avoidance/reducing exposure to cues for the behavior (12.3); Goal setting (behavior) (1.1)
Negative thoughts domain of LBM	Psychoeducation on impact on negative thoughts; Mind traps; Cognitive restructuring; Challenge thoughts that may be unhelpful	Psychoeducation; Guided self-help; International Treatment Effectiveness Project (ITEP); Cognitive-behavioral therapy (CBT)	Information about antecedents (4.2); Information about health consequences (5.1); Salience of consequences (5.2); Information about social and environmental consequences (5.3); Information about emotional consequences (5.6); Re-attribution (4.3); Framing-reframing (13.2)
Emotions domain of LBM	Psychoeducation on impact on emotions; Attention narrowing; Attention switching; Emotional regulation; Recognize/understand/normalize emotions; Developing more appropriate coping strategies	Psychoeducation; Guided self-help; Coping strategy enhancement (CSE); Mindfulness-based cognitive therapy	Information about antecedents (4.2); Information about health consequences (5.1); Salience of consequences (5.2); Information about social and environmental consequences (5.3); Information about emotional consequences (5.6); behavioral practice/rehearsal (8.1); Reduce negative emotions (11.2); Problem solving (1.2); Social support (unspecified) (3.1); behavioral practice/rehearsal (8.1); Distraction (12.4)
Physical sensations domain of LBM	Psychoeducation on impact of physical sensations; Urge surfing; Body scanning; Relapse prevention-based techniques	Psychoeducation; Guided self-help; Mindfulness-based cognitive therapy	Information about antecedents (4.2); Information about health consequences (5.1); Salience of consequences (5.2); Information about social and environmental consequences (5.3); Information about emotional consequences (5.6); Instruction on how to perform a behavior (4.1); behavioral practice/rehearsal (8.1); Reduce negative emotions (11.2)
Unhelpful behaviors domain of LBM	Psychoeducation on impact of destructive behaviors; Activity scheduling; Behavioral activation; Encourage new behaviors via positive feedback; Increase activity to increase energy levels and relieve boredom	Psychoeducation; Guided self-help; Cognitive-behavioral therapy (CBT)	Information about antecedents (4.2); Information about health consequences (5.1); Salience of consequences (5.2); Information about social and environmental consequences (5.3); Information about emotional consequences (5.6); Non-specific reward (10.3); Non-specific incentive (10.6); Reward approximation (14.4); Rewarding completion (14.5); Goal setting (behavior) (1.1); Action planning (1.4)
Lifestyle domain of LBM	Psychoeducation on impact of lifestyle; Creating SMART goals for recovery; Goalsetting Increase treatment engagement and retention. Increase readiness to change behavior	Psychoeducation; Guided self-help; Motivational enhancement therapy (MET); Implementation intentions	Goal setting (behavior) (1.1); Problem solving (1.2); Goal setting (outcome) (1.3); Action planning (1.4); Non-specific reward (10.3); Focus on past success (15.3)

### Relevant concomitant care permitted or prohibited during the trial

The aim of this study is to examine effectiveness of BFO when delivered via a telehealth approach as an adjunct to standard treatment, therefore, both interventional and control groups will receive standard treatment. Existing standard treatment utilized by the participating NHS Trust may be variable, therefore it is expected that there will be a degree of heterogeneity within each of the study groups—this heterogeneity will be captured in the participant’s source data. Treatments usually available in outpatient SUD services include standard low-intensity interventions such as motivational and engagement tools to reduce substance use—more intensive psychological therapies such as CBTs may also be delivered. Not all standard treatment sessions will be delivered in-person—some participants may receive sessions via telephone calls rather than in-person sessions in the service. Standard treatment sessions will likely have a duration range of 30–60 min and will take place once or twice a week for eight-weeks. Medications may also be prescribed, including substitute medications such suboxone, buprenorphine etc. and also psychotropic medications such as anti-depressants, anti-anxiolytics etc.

### Outcomes

Outcomes data will be collected via self-report, with participants completing an online assessment containing a series of standardized psychometric measures at Baseline, End of Treatment, and 3-month and 6-month follow up timepoints. Additionally, engagement data (time spent accessing the program, number of BCTs engaged with) will be collected automatically via the BFO backend database for those participants in the interventional arm.

The primary outcome will be self-reported substance use compared to baseline following treatment completion, and at three and six-months follow-up, which will be measured using the following questions:

- Weekly use of primary problem substance (i.e., ‘How many days in the past week did you use [primary substance]?’
- ‘How much [primary substance] did you use each day?’)

Secondary outcomes will be measured at corresponding time points using the following standardized measures:

- Severity of Dependence Scale (SDS) [37, 38]: A five-item scale measuring severity of substance dependence—internal reliability: α = 0.81–0.90; test–retest reliability ICC = 0.89.
- Patient Health Questionnaire-4 (PHQ-4) [39]: A four-item scale that measures severity of depression and anxiety—internal reliability, α = 0.81.
- Five items (1, 2, 17, 18, 20) from the World Health Organization Quality of Life scale (WHOQoL-BREF) [40]—internal reliability of these five items, α = 0.84.
- Recovery Progression Measure (RPM) [34, 35]: A 36-item scale measuring functioning in six biopsychosocial domains functioning that are implicated in all SUDs and recovery from SUDs—internal consistency, α = 0.89; test–retest reliability, ICC = 0.73.

### Randomization

Randomization will occur at the level of individual participants using a random allocation sequence via the Research Randomizer [41]. Sequentially numbered opaque sealed envelopes containing the group that each participant will be allocated to will be prepared prior to commencement of the study. Treatment allocation sequences will be generated by a member of the research team and disseminated to the participating organization via sealed opaque envelope. Participants will be enrolled into the study and subsequently assigned to a treatment condition by a member of the research team at the participating organization. Because this study is a non-pharmaceutical study open-label study, no blinding is required—however, the study statistician will be blinded to treatment allocation when analyzing study data. Participants will be identified by a participant identification or randomization number only on all trial documentation, except for informed consent documents. Where there is a need for Sponsor personnel to verify trial documentation against medical records or other data sources containing identifiable information, this will be permitted by the research site, provided that subject confidentiality is maintained in accordance with local regulations and requirements.

### Adverse event reporting

Historically, the recording and reporting of adverse events (AEs) has been inconsistent and poorly defined in psychological and behavioral intervention randomized controlled trials (RCTs) [42]. One factor that may contribute to the under-reporting of adverse events in such trials is the assumption that AEs are purely medical in nature and are thus unlikely to be caused by non-medicinal interventions. However, psychological and behavioral interventions may cause psychosocial harms [43]. Behavioral interventions requiring individuals to reflect on their substance use may cause non-medical adverse events, or ‘social AEs’ [44] such as involvement with law enforcement, safeguarding referrals, perpetration of domestic abuse, and involvement with social services. These alternative harms are often poorly documented in the context of RCTs [45], yet may prove vital when assessing the risk–benefit profile of an intervention. In addition to this, the use of computers to deliver behavioral interventions may mean an increased incidence of technology-related medical adverse events, such as increased incidences of headaches and migraines, which may feasibly be medical consequences of a non-medicinal intervention.

Both medical and social AEs will be documented and monitored throughout the course of the study for both groups. Adverse event data will be obtained via retrospective review of participants’ medical notes—events occurring between randomization and the 6-month follow-up timepoint will be recorded at the time of each participant’s completion of the study.

### Criteria for discontinuing or modifying allocated interventions

Participants may be discontinued from BFO or standard treatment interventions at their own request at any point during the study. Additionally, participants’ standard treatment may be modified at the discretion of the Principal Investigator and clinical team at the participating site in response to worsening health status, or in response to perceived or actual harms resulting from their health status. Any discontinuation or modification of allocated interventions will be documented appropriately.

### Provisions for post-trial care

All patients will be returned to standard care within the participating Trust after inclusion, facilitated by the Trust’s research team and site PI. If no negative effects or serious adverse events are found to be associated with BFO, all participants, regardless of treatment allocation during the study, will be offered access to the program after trial inclusion.

### Participant timeline

**Fig. 2:**
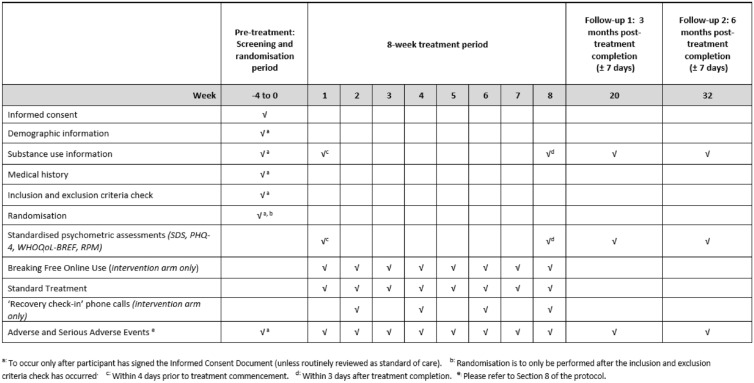
Study timeline and participant activity schedule

Both groups will complete a period of eight-weeks of SUD treatment, of either (i) BFO plus standard treatment, or (ii) standard treatment only. The assessment battery will then be administered to all participants, via an online or hardcopy version, at the end of the eight-week treatment period, and at three and six-months follow-up. Each participant will take part in the study for a total of approximately 10-months. Please see Fig. 2 for a full description of the study timeline and all participant activities, including treatment schedule and assessment timepoints. 

### Data analysis and management

Data will be analyzed using SPSS® Version 26.0 (or later). The principal data analytical strategy will be a repeated measures analysis of variance (ANOVA) in order to make a longitudinal comparison of treatment groups for the primary and secondary outcomes along the follow-up time points. The appropriate 95% confidence interval will be applied. The main statistical analyses will be conducted by the Chief Investigator with specialist statistical support from co-investigators at the collaborating academic institution (University of Manchester). Main analyses based on published research [26] indicate that data will likely be non-normally distributed, and that therefore, the most appropriate analyses will be Kruskall-Wallis ANOVA and Analyses of Covariance (ANCOVA) at each psychometric assessment time point, in order to compare study groups.

Changes over time in psychometric assessment scores within each group will be conducted using Wilcoxon Signed Ranks Tests—a more conservative significance level of p = 0.001 will be adopted to compensate for the increased risk of Type I errors associated with multiple comparisons. Effect sizes will also be calculated to examine robustness of between-group differences and within-group changes, in addition to examining clinically significant changes over time by analyses of numbers of participants fulfilling clinical threshold scores for substance dependence, depression and anxiety. Regression analyses will be conducted to control for baseline differences between groups in terms of sample size, severity of substance dependence and mental health sequelae, psychosocial functioning and quality of life, as measured by the battery of standardized psychometric assessments. Additionally, mixed effects models will be used to compensate for the independence assumption inherent to regression analyses.

Interim analyses will be performed on the first 30 participants of each group to have completed the three- and six-months follow-up assessments after treatment completion. The data will be subjected to ANOVA and ANCOVA. Changes over time in psychometric assessment scores within each group will be conducted using Wilcoxon Signed Ranks Tests, assuming data are non-normally distributed. If following the interim analysis, a significant difference in outcomes is detected between the groups, either the sample size will be recalculated or recruitment will be discontinued, depending on the statistical power obtained.

Sub-group analyses will be conducted to examine the influence of sociodemographic variables such as age and gender, and clinical characteristics such as primary problem substance and clinically relevant depression and anxiety, on outcomes. Sub-group analyses will be conducted using ANOVA, ANCOVA and regression.

Intention-to-treat (ITT) principles will be followed, with data from all randomised participants being included in these analyses. Mixed effect models will be used to handle missing data by maximum likelihood estimation, which uses all available data from randomised participants and has been demonstrated to minimise bias more effectively than other approaches [46]. A number of strategies will be employed to maximize follow-up and prevent missing data as far as possible. These will include the study team contacting participants directly to chase-up incomplete assessments and working closely with clinical staff to contact those participants who cannot be contacted by the study team directly—reasons for missing data and drop-out will be recorded where this information is available. Additionally, non-randomised, observational, per-protocol comparative analyses will be conducted using data only from participants who fulfilled all protocol requirements in terms of eligibility, intervention receipt, and outcome assessment completion, in order to ascertain effects from BFO under ideal conditions.

Responsibility for data management will lie with the Chief Investigator, and the Principal Investigator within the Sponsor organization. A detailed allocation of responsibilities will be completed by the Chief Investigator as a separate document (‘Delegation Log’), which will be followed by the investigational site personnel and Sponsor personnel. Routine data monitoring for this trial will be completed by the Sponsor Clinical Trial Coordinator, who will conduct monitoring visits on a pre-determined schedule with the site Principal Investigator, other relevant clinicians at site, and trial staff. The Clinical Trial Coordinator will report directly to the Chief Investigator and Sponsor organization. Monitoring and auditing intervals for this study will be based on a risk-assessment undertaken by the Sponsor prior to first participant recruitment and will be appropriately communicated to the study site in advance. The trial will adhere to any routine internal audit processes already in place within the participating organization.

## Discussion

This protocol describes the methodology for an RCT to examine the effectiveness of digital CBT for SUD—Breaking Free Online (BFO)—and fortnightly recovery check-in phone calls, when delivered as an adjunct to standard treatment, compared with standard treatment only, at an NHS Mental Health Trust in North-West England. This design could be interpreted as a limitation as it does not allow direct effectiveness comparisons to be made between digital treatment and in-person treatment. However, this design has been selected in order to enhance ecological validity of the study, as it most accurately reflects how BFO is delivered in treatment services, i.e., as an adjunct to standard treatments and not as a replacement.

Another limitation of the methodology is that it will not be possible for the investigators and practitioners to be blinded to the allocation of participants, which could potentially bias outcomes—however, the study statistician will be blinded to treatment allocation. Additionally, because the level of randomization will be at the level of individual service users, this may pose a risk of contamination across the two study groups if individual participants have an opportunity to interact with one another. Participants may also join the study at different points in their treatment and recovery journey—therefore a ‘floor effect’ could occur that may under-estimate the effectiveness of BFO if a proportion of participants start at a baseline of abstinence/low substance use and therefore relatively good psychosocial functioning.

Another potential limitation is the likely attrition rate from a study including individuals with SUD – previous research has demonstrated high levels of attrition in SUD intervention RCTs, especially when the intervention under investigation is digital [47, 48]. Individuals with SUD may have mental health difficulties, financial and accommodation instability, and may experience periods of lapse and relapse; each of these factors may create difficulties when trying to support such individuals to remain engaged with treatment programs [49, 50]. Although the fortnightly recovery check-in phone calls are intended to enhance retention of participants in the BFO arm of the study, it may be more challenging to retain participants in the study once the treatment period is over and they are no longer receiving the check-in phone calls—a prediction supported by previous systematic reviews into smoking cessation interventions [51]. Every attempt will be made to obtain follow-up data—as follow-up data is being collected via electronic methods, there is scope to send reminders to participants to complete follow-up assessments via email, a method indicated to be successful in increasing follow-up retention in previously conducted RCTs [52]. 

Despite the potential limitations of a study such as this, if BFO and the telehealth delivery approach included appear to support individuals to achieve recovery from SUD, findings could inform treatment delivery across SUD services more generally and could increase patient access to support even when in-person services may be restricted or unavailable.

## Data Availability

Not applicable.

## Abbreviations

AE: Adverse event
ANCOVA: Analysis of covariance
ANOVA: Analysis of variance
BCT: Behavioral change technique
BFO: Breaking free online
CAT: Computer-assisted therapy
GMMH: Greater Manchester mental health
ITT: Intention to treat
LBM: Lifestyle balance model
NHS: National Health Service
PHQ-4: Patient health questionnaire-4
RCT: Randomized controlled trial
RPM: Recovery progression measure
SDS: Severity of dependence scale
SPSS: Statistical package for the social sciences
SUD: Substance use disorders
ITT: Intention to treat
WHOQoL-BREF: World Health Organization Quality of Life Scale
